# Multifocal Low‐Grade Oncocytic Tumor of the Kidney Associated With Germline *TSC1* Alteration: Case Report and Literature Review

**DOI:** 10.1155/crip/4811653

**Published:** 2026-07-16

**Authors:** Ilham Putra, Tanishq Vashisht, Atousa Ordobazari, Aram Vosoughi

**Affiliations:** ^1^ Department of Pathology, H Lee Moffitt Cancer Center and Research Institute, Tampa, Florida, USA, moffitt.org

**Keywords:** germline, LOT, low-grade oncocytic tumor, multifocal, *TSC1*

## Abstract

Low‐grade oncocytic tumor (LOT) of the kidney is a rare, indolent neoplasm within the spectrum of eosinophilic renal tumors. It typically presents as a small, solitary mass and is associated with molecular alterations in *TSC1*, *TSC2*, or *MTOR*. We describe a 72‐year‐old male with a history of pancreatic neuroendocrine tumor, gastrointestinal stromal tumor, and prostate cancer who presented with multiple bilateral renal masses. Partial nephrectomy revealed two distinct tumors: one consistent with LOT and the other with a sclerosing angiomyolipoma (AML). The LOT consisted of oncocytic cells with round to oval nuclei and delicate perinuclear halos, arranged predominantly in solid architecture, and immunoreactive for PAX8, EMA, and CK7, whereas negative for CD117 and AMACR. The AML was composed of spindle and epithelioid cells embedded in sclerotic stroma, positive for MiTF and SMA, and negative for conventional melanocytic markers, including HMB45 and Melan‐A. Germline testing identified a *TSC1* intron 6, c.509‐15G > A variant of uncertain significance. Somatic analysis showed increased allelic imbalance at the *TSC1* locus, suggesting loss of heterozygosity and a potential pathogenic role. This case adds to the limited reports of multifocal LOT and demonstrates the consistent association of germline *TSC1* alterations with this rare tumor presentation.

## 1. Introduction

The application of immunohistochemistry and molecular tests for characterizing renal tumors has significantly evolved, aiding in the recognition and differentiation of renal tumors, including those with oncocytic features. Despite these advancements, accurate classification remains challenging due to considerable morphologic and immunophenotypic overlap among renal oncocytic tumors. The spectrum of renal oncocytic neoplasms includes epithelioid angiomyolipoma (AML), succinate dehydrogenase–deficient renal cell carcinoma (RCC), fumarate hydratase–deficient RCC, MiT family gene–rearranged RCC, eosinophilic solid and cystic RCC (ESC‐RCC), as well as two newly recognized entities—low‐grade oncocytic tumor (LOT) and eosinophilic vacuolated tumor (EVT) [1, 2]. LOT and EVT have intermediate features that position them between conventional oncocytoma and chromophobe RCC.

ESC‐RCC, EVT, and LOT are commonly associated with genetic alterations involving the mTOR pathway (*TSC1*, *TSC2*, or *MTOR*) [1–5] . LOT is typically a solitary tumor and is commonly associated with sporadic somatic mutations involving the mTOR signaling pathway, particularly activating mutations in *MTOR* or inactivating mutations in *TSC1* or *TSC2* [1, 3–6]. Less commonly, LOT may occur in association with germline mutations in *TSC1* or *TSC2* and can be multifocal, typically in the context of tuberous sclerosis complex (TSC) [4, 5, 7]. Renal AMLs are the most frequent renal manifestation of TSC [8]. Additionally, *TSC1* and *TSC2* mutations have also been associated with pancreatic neuroendocrine tumors (Pan‐NETs) [9].

LOT, as defined by the Genitourinary Pathology Society (GUPS), usually occurs as a solitary, nonsyndromic lesion in older patients, with a median age of 67 years, a slight female predominance, and typically follows an indolent clinical course [1]. Gross examination typically reveals a small (approximately 3–4 cm), solid, tan‐yellow to brown mass. Histologically, LOT is characterized by granular eosinophilic cells with uniform round‐to‐oval nuclei, delicate perinuclear halos, and an absence of significant nuclear irregularities. Immunohistochemically, LOT exhibits a distinctive profile, notably CK7 positivity and CD117 negativity, allowing differentiation from classic oncocytoma and chromophobe RCC [1, 2]. This report describes a rare presentation of multifocal LOT, including detailed germline and somatic genetic analyses, underscoring the significance of *TSC1* genetic alterations in such cases.

## 2. Case Presentation

A 76‐year‐old male was incidentally found to have multiple small bilateral renal masses involving the upper and lower poles of both kidneys, the largest measuring 1.7 cm. His past medical history included a Grade 1 Pan‐NET, gastric gastrointestinal stromal tumor (GIST), and Gleason 3 + 4 (Grade Group 2) prostatic adenocarcinoma. Follow‐up imaging 18 months later showed interval growth of the largest mass to 2.5 cm. The patient underwent right partial nephrectomy of both upper and lower pole masses.

## 3. Histopathology

**Figure 1:**
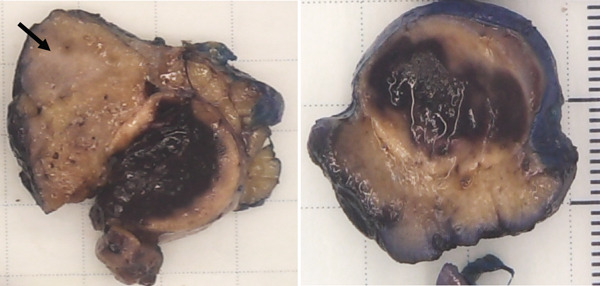
Partial nephrectomy specimens showing multifocal, well‐circumscribed, nonencapsulated renal masses with focal hemorrhage, consistent with low‐grade oncocytic tumors (LOT). An adjacent ill‐defined, white fibrotic lesion (arrow) corresponds to sclerosing angiomyolipoma.

Macroscopic examination revealed a well‐circumscribed, nonencapsulated mass displaying a mahogany‐brown to yellow‐tan cut surface and focal hemorrhagic areas. Adjacent to this mass, an ill‐defined, white‐tan fibrotic region was noted (Figure 1). Microscopically, two distinct neoplasms were identified. The first tumor comprised oncocytic cells exhibiting finely granular, eosinophilic cytoplasm, uniform round‐to‐oval nuclei, and subtle perinuclear halos. These cells were arranged predominantly in a solid and nested architectural pattern.

**Figure 2:**
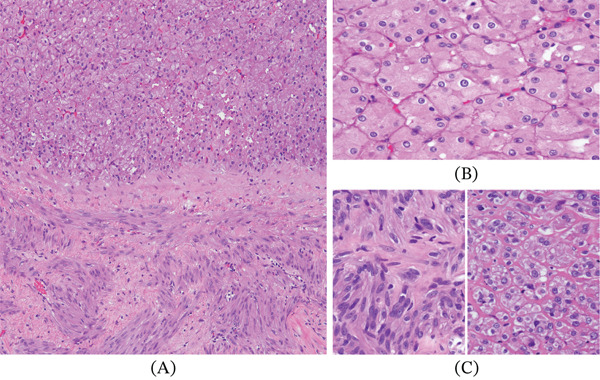
Two adjacent renal neoplasms with distinct morphologic features were identified (2A). The low‐grade oncocytic tumor (LOT) is composed of oncocytic cells with uniform round‐to‐oval nuclei and perinuclear halos, predominantly arranged in a solid architectural pattern (2B). The sclerosing angiomyolipoma consists of spindle cells with eosinophilic cytoplasm arranged in fascicular patterns, admixed with scattered epithelioid cells within a sclerotic stroma (2C).

**Figure 3:**
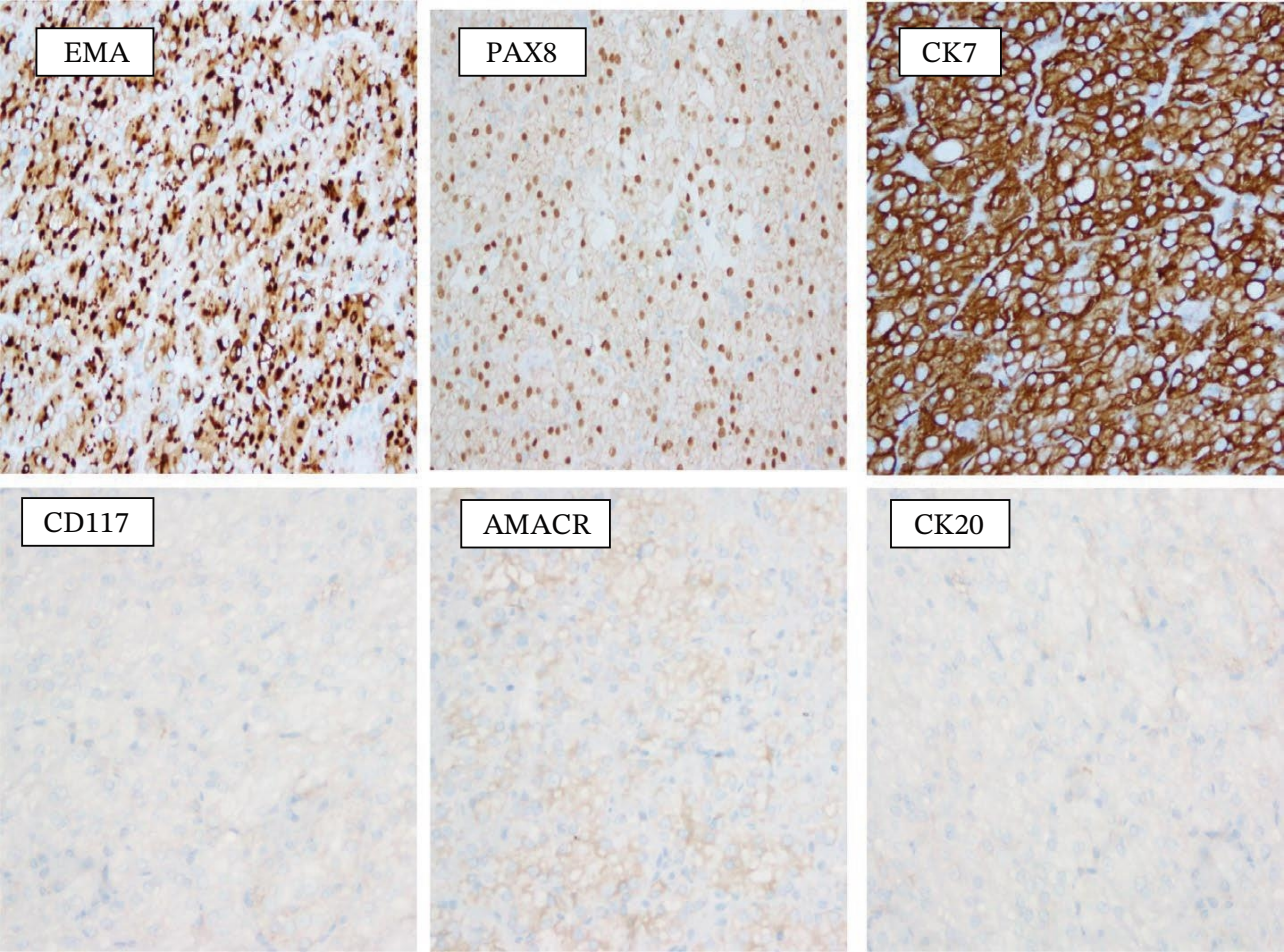
Immunohistochemical stains of low‐grade oncocytic tumor (LOT).

**Figure 4:**
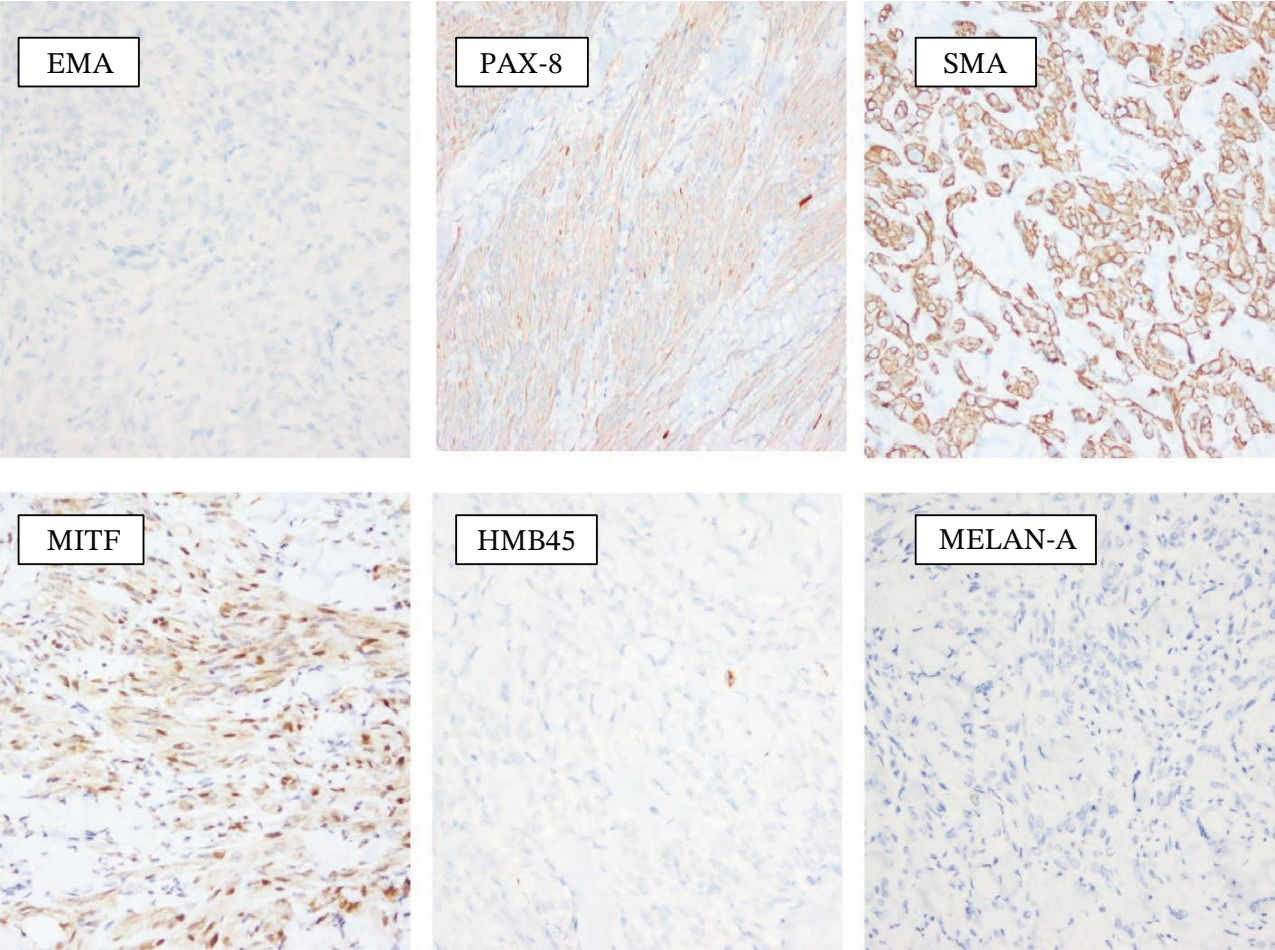
Immunohistochemical stains of sclerosing angiomyolipoma.

The second lesion comprised uniform spindle cells with eosinophilic cytoplasm arranged in fascicular patterns, accompanied by scattered epithelioid cells embedded within a sclerotic and hyalinized stroma (Figure 2). Neither neoplasm exhibited nuclear atypia, mitotic activity, or necrosis. Immunohistochemical analysis demonstrated diffuse positivity for EMA, PAX8, and CK7 in the oncocytic tumor cells, and negativity for CD117, AMACR, and CK20, supporting the diagnosis of LOT (Figure 3). In contrast, the spindle cell lesion exhibited immunoreactivity for SMA and MiTF while lacking expression of EMA, PAX8, Melan‐A, and HMB45, supporting the diagnosis of the rare sclerosing variant of AML (Figure 4) [2, 8].

## 4. Molecular Studies

Germline next‐generation sequencing (NGS) analysis employing a comprehensive 156‐gene cancer panel (InVitae), which includes high‐risk inherited cancer genes associated with various malignancies, identified a *TSC1* intronic variant (intron 6, c.509‐15G > A) with a variant allele frequency (VAF) of 42% in peripheral blood. Subsequent targeted somatic NGS (170‐gene panel; Moffitt STAR) performed on tumor tissues (LOT and sclerosing AML) also detected the same *TSC*1 intronic/splice variant, demonstrating an increased VAF of 63%. No other pathogenic somatic mutations were identified. The higher VAF observed in the tumor compared to peripheral blood suggests loss of heterozygosity at the *TSC1* locus, supporting a potential pathogenic role for this variant, despite its current classification in ClinVar as a variant of uncertain significance or likely benign.

## 5. Discussion

Recent molecular studies have significantly advanced our understanding and classification of renal tumors, including those with oncocytic characteristics. Several entities with morphologic features intermediate between renal oncocytoma and chromophobe RCC have been identified, including ESC‐RCC, EVT, and LOT [1, 2]. These tumors frequently harbor genetic alterations involving the mTOR pathway, particularly mutations in *TSC1*, *TSC2*, or *MTOR*] [1–6, 10].

LOT, recognized as an emerging entity in the 2022 WHO classification, represents less than 0.5% of all renal tumors yet is comparatively common among renal oncocytic neoplasms. It typically presents as a solitary lesion with a characteristic immunoprofile—diffuse positivity for CK7 and negativity for CD117—which aids differentiation from renal oncocytoma and chromophobe RCC [2]. Multifocal or bilateral LOT presentations associated with germline mutations are rare, highlighting the necessity of comprehensive germline and somatic analyses to better understand their molecular basis [4, 5, 10].

In this report, we describe a rare case of multifocal LOT occurring simultaneously with multifocal sclerosing AML, a rare AML variant characterized by prominent sclerosis with minimal or absent adipocyte differentiation. Our patient, a 76‐year‐old male, had a complex oncologic history, including PanNET, GIST, and prostatic adenocarcinoma.

Histopathologic examination confirmed LOT with oncocytic renal tumor cells characterized by uniform round‐to‐oval nuclei, subtle perinuclear halos, and minimal nuclear atypia arranged predominantly in a solid pattern. Immunohistochemical analysis confirmed the diagnosis with diffuse CK7 and PAX8 positivity, and negativity for CD117, AMACR, and CK20. Recently described immunohistochemical markers provide additional supportive value in the diagnosis of LOT. Glycoprotein nonmetastatic melanoma protein B (GPNMB) is a useful marker in the diagnosis of *TSC1/TSC2/MTOR* alteration–associated renal neoplasms, including LOT [11]. In addition, immunostaining for L1 cell adhesion molecule (L1CAM), a marker of principal cells of the collecting ducts, along with GATA3, is consistently positive in LOT and can aid in distinguishing it from other oncocytic renal tumors, particularly eosinophilic chromophobe RCC [6, 12].

The adjacent sclerosing AML showed spindle and epithelioid cells within a densely hyalinized stroma, positive for MiTF but negative for melanocytic markers (HMB45, Melan‐A) [2].

Comprehensive molecular analyses revealed a germline *TSC1* intron 6 (c.509‐15G > A) variant with a VAF of 42%. This variant is listed in ClinVar as of uncertain significance or likely benign. Notably, the same variant was detected in both LOT and AML tumors with a higher VAF of 63%, raising the possibility of allelic imbalance or loss of heterozygosity at the *TSC1* locus and suggesting a potential pathogenic role in tumorigenesis, particularly given the absence of other pathogenic mutations

A literature review (summarized in Table 1) identifies additional cases of multifocal LOT, showing a slight male predominance and age range from 43 to 76 years [4, 5, 10]. Only one case exhibited classic TSC manifestations, consistent with prior studies showing that patients with TSC, particularly those harboring *TSC1* alterations, may exhibit a milder clinical phenotype and variable disease expressivity [13]. End‐stage renal disease was rare in this cohort. Our patient′s unique combination of tumors—PanNET, GIST, and prostate adenocarcinoma—has not been previously reported alongside multifocal LOT, emphasizing the distinctiveness of this presentation. Germline mutations in *TSC1* or *TSC2* are associated with PanNETs, which typically present at a younger age and may be multifocal as well as with rare GIST with wild‐type *KIT* and *PDGFRA* status [9, 14]. Additionally, germline *TSC* mutations have been suggested to increase the risk of certain genitourinary malignancies including prostate cancer [15].

Among reported cases of multifocal LOT, AML was the most common concurrent neoplasm, identified in three out of five patients. Bilateral renal involvement was noted in three cases, with tumor sizes ranging from 0.2 to 5.3 cm. The highest reported tumor stage among these multifocal LOT lesions was pT3ab [10].

**Table 1:** Clinical, pathological, and molecular characteristics of reported multifocal low‐grade oncocytic tumors (LOT).

Age/sex	Past medical history	Family history	Renal tumor type and number	Number and laterality of LOT lesions	Genomic alteration (germline/somatic)	Tumor size LOT (cm)	Tumor stage (highest stage of concurrent tumors)	Reference
63/F	N/A	Father with B/L nephrectomies for unknown reason	B/L multiple LOTs	6 LOTs (RK), 10 LOTs (LK)	Germline—TSC1 c.2208+2 T > A Somatic—TSC1 c.2273_2276dup	0.5–5.3	RK—pT1a^a^ LK—pT1b^a^	(Kapur et al.) [4]
43/M	Skin hamartomas, multiple cortical tubers	N/A	Unilateral LOTs	2 LOTs (LK)	Somatic—TSC1 c.2549_2552dup (also present in benign tissue)	1.7, 2.1	(not mentioned)	(Ricci et al.) [5]
49/M	ESRD (FSGS) Retinal detachment Hyperparathyroidism	Sibling: intestinal cancer Father: pancreatic cancer Mother: breast cancer	Unilateral LOTs, 2 ESC‐RCCs, 1 EVT, Multiple AMLs, 1 RCC, NOS	3 LOTs (RK)	Germline—TSC1 c.2074C > (p.Arg 692∗)	0.2–0.5	RK—pT1a^a^	(Lerma et al.) [10]
49/M	None	No first‐ or second‐degree relatives with CA	B/L LOTs, 1 RCC‐FMS, 3 AMLECs, 2 RCC‐FMSs	7 LOTs (RK), 2 LOTs (LK)	Germline—TSC1 c.395_406del ins CA (p.Gly 132Alafs∗2)	0.3–5	RK—pT3a^a^ LK—pT1b^b^	(Lerma et al.) [10]
76/F	GIST, pancreatic NET, prostate cancer	N/A	B/L LOTs, Multiple AMLs	2 LOTs (RK), 1 LOT (LK)	Germline and somatic—TSC1 intron 6, c.509‐15G > A alteration	2.5, 2.2, 0.6	pT1a	Current case

Abbreviations: AMLEC, angiomyolipoma with epithelial cysts; B/L, bilateral; ESC‐RCC, eosinophilic solid and cyst RCC; ESRD, end stage renal disease; EVT, eosinophilic vacuolated tumor; FSGS, focal segmental glomerulosclerosis; GIST, gastrointestinal stromal tumor; LK, left kidney; LOT, low‐grade oncocytic tumor; NET, neuroendocrine tumor; RCC‐FMS, RCC with fibromyomatous stroma; RCC, NOS, RCC not otherwise specified; RK, right kidney; TSC, tuberous sclerosis.

^a^pT—represents highest stage of LOT.

^b^pT—represents highest stage of RCC‐FMS.

Additionally, although *MTOR* mutations are commonly reported in LOT, multifocal LOT cases show a distinctive and strong association with germline *TSC1* mutations [4, 5, 10].

In summary, we report a rare case of multifocal bilateral LOT occurring in a patient who presented with multiple distinct renal and extrarenal tumors but lacked the classic clinical manifestations of TSC. The identification of *TSC1* intronic variant as the sole significant genomic alteration in both germline and somatic analyses highlights its potential pathogenic significance in this multifocal LOT case. This finding underscores the importance of comprehensive genetic evaluations in rare tumor presentations, particularly when an underlying hereditary component is suspected.

Furthermore, a review of molecular analyses from previously reported multifocal LOT cases consistently demonstrates the shared germline *TSC1* genomic alteration among these tumors.

## Author Contributions

Conceptualization: I.P. and A.V. Data acquisition: I.P., T.V., A.O., and A.V. Formal analysis: I.P. and A.V. Writing—original draft: I.P., A.O., and A.V. Writing—review and editing: I.P., A.O., and A.V.

## Funding

No funding was received for this manuscript.

## Disclosure

All authors have read and agreed to the published version of the manuscript.

## Ethics Statement

This study was conducted in accordance and compliance with all statutes, directives, and guidelines of an Internal Review Board authorization involving retrospective data analysis, which did not require informed consent.

## Consent

The authors have nothing to report.

## Conflicts of Interest

The authors declare no conflicts of interest.

## Data Availability

The data that support the findings of this study are available from the corresponding author upon reasonable request.
